# Emergence of Polymorphic Mating Strategies in Robot Colonies

**DOI:** 10.1371/journal.pone.0093622

**Published:** 2014-04-09

**Authors:** Stefan Elfwing, Kenji Doya

**Affiliations:** 1 Neural Computation Unit, Okinawa Institute of Science and Technology Graduate University, Okinawa, Japan; UC Santa Barbara, United States of America

## Abstract

Polymorphism has fascinated evolutionary biologists since the time of Darwin. Biologists have observed discrete alternative mating strategies in many different species. In this study, we demonstrate that polymorphic mating strategies can emerge in a colony of hermaphrodite robots. We used a survival and reproduction task where the robots maintained their energy levels by capturing energy sources and physically exchanged genotypes for the reproduction of offspring. The reproductive success was dependent on the individuals' energy levels, which created a natural trade-off between the time invested in maintaining a high energy level and the time invested in attracting mating partners. We performed experiments in environments with different density of energy sources and observed a variety in the mating behavior when a robot could see both an energy source and a potential mating partner. The individuals could be classified into two phenotypes: 1) *forager*, who always chooses to capture energy sources, and 2) *tracker*, who keeps track of potential mating partners if its energy level is above a threshold. In four out of the seven highest fitness populations in different environments, we found subpopulations with distinct differences in genotype and in behavioral phenotype. We analyzed the fitnesses of the foragers and the trackers by sampling them from each subpopulation and mixing with different ratios in a population. The fitness curves for the two subpopulations crossed at about 25% of foragers in the population, showing the evolutionary stability of the polymorphism. In one of those polymorphic populations, the trackers were further split into two subpopulations: (*strong trackers*) and (*weak trackers*). Our analyses show that the population consisting of three phenotypes also constituted several stable polymorphic evolutionarily stable states. To our knowledge, our study is the first to demonstrate the emergence of polymorphic evolutionarily stable strategies within a robot evolution framework.

## Introduction

If you come to any more conclusions about polymorphism, I should be very glad to hear the result: it is delightful to have many points fermenting in one's brain, and your letters and conclusions always give one plenty of this same fermentation.

- Charles Darwin, letter to Joseph Hooker, 1846 [1]

Polymorphism has fascinated evolutionary biologists since the time of Darwin [2], [3]. Polymorphism is defined as that there exist more than one distinct phenotype of a species occupying the same habitat at the same time [4], [5]. Polymorphism does not include continuous variations, but only discrete variations or in the case of continuous traits, such as body size and color, strongly bimodal or multimodal phenotype variation distributions. The existence of more than one distinct phenotype of a species demands an explanation, because the theory of natural selection predicts that the fittest phenotype should drive the other, lesser fit phenotypes to extinction. In general, polymorphism is maintained if the “fitness curves” of the polymorphic phenotypes intersect, where the crossover-point is an evolutionarily stable state, realizing a polymorphic evolutionarily stable strategy (ESS) [6], [7]. Common features of the evolution and the maintenance of behavioral polymorphism include: 1) that time or resources can be invested in more than one activity that contributes to the fitness; 2) that the individuals have rules about how to allocate time and resources among the alternative activities; and 3) that there is a trade-off between the activities that contribute to the fitness, i.e., the allocation of time and resources invested in one activity could be invested in another [8]. Frequency-dependent selection [7], [9], [10] is considered the most important explanation for the maintenance of polymorphism in a population. Frequency-dependent selection occurs when the fitness of the phenotypes depends on their frequencies in the population, and the fitness curves intersect at a crossover frequency where the phenotypes are equally successful. Alternative mating strategies (or alternative reproductive behaviors) [11]–[13] is the area of biological research most closely related to this study. Different mating tactics has been observed in a wide variety of species, both in males (e.g., [14]–[16]) and in females (e.g., [17]–[19]). However, there are relatively few cases where the differences in mating behavior have been confirmed to have a genetic basis [20]–[26], and even fewer studies that have suggested equal average reproductive success, i.e., shown crossing of the fitness curves, of alternative phenotypes [21], [22], [27].

The use of robot evolution experiments to study biological phenomena has gained traction in recent years [28], as a complementary approach to biological studies and theoretical models. In comparison to biological studies, robot evolution has the advantage that the evolution of hundreds of generations of robot controllers can be completed within hours or days. The experiments can easily be repeated for different parameter settings and environmental conditions, which allows for quantitative testing and analysis of robustness and stability. In comparison with theoretical and numerical models, robot models can capture the often complex physical interactions between the agent and the environment, including other agents. Floreano and Keller with different co-authors have used robot evolution experiments to investigate the emergence and reliability of communication [29]–[32], to quantitatively test Hamilton's rule for the evolution of altruism [33], and to test the influence of genetic architecture and mating frequency on the division of labor in social insect societies [34].

A distinctive feature of our earlier proposed embodied evolution framework [35] is that there is no explicit fitness function or algorithm for selecting individuals for recombination and mutations. Instead, offspring can only be created by the physical exchange of genotypes between two mating robots. In general, the choice of selection method requires careful consideration when using artificial evolution to study ESSs. A strong theoretical assumption underlying ESS analysis is that the population is infinitely large. Fogel *et al.* [36]–[38] demonstrated in simulation experiments, using the Hawk-Dove game, that for finite populations the results differed, at best, significantly from the theoretical ESS values and, at worst, bore no resemblance to the ESS. They, therefore, questioned the usefulness of ESSs to explain real biological phenomena in populations with limited population sizes. In response, Ficici and Pollock [39] showed that the difference between the theoretical ESS predictions and the observed simulation results could be accounted by the two selection methods used by Fogel *et al.*.

The purpose of this study is to demonstrate that evolutionary stable alternative mating strategies can emerge in a small robot colony without any predefined mating preferences as a result of the trade-off between the resources spent on energy conservation and the resources spent on courtship of mating partners. As alternative mating strategies is a natural precursor for the evolution of sexual dimorphism, this line of research has the potential of increasing our understanding of the emergence of different sexes.

**Figure 1:**
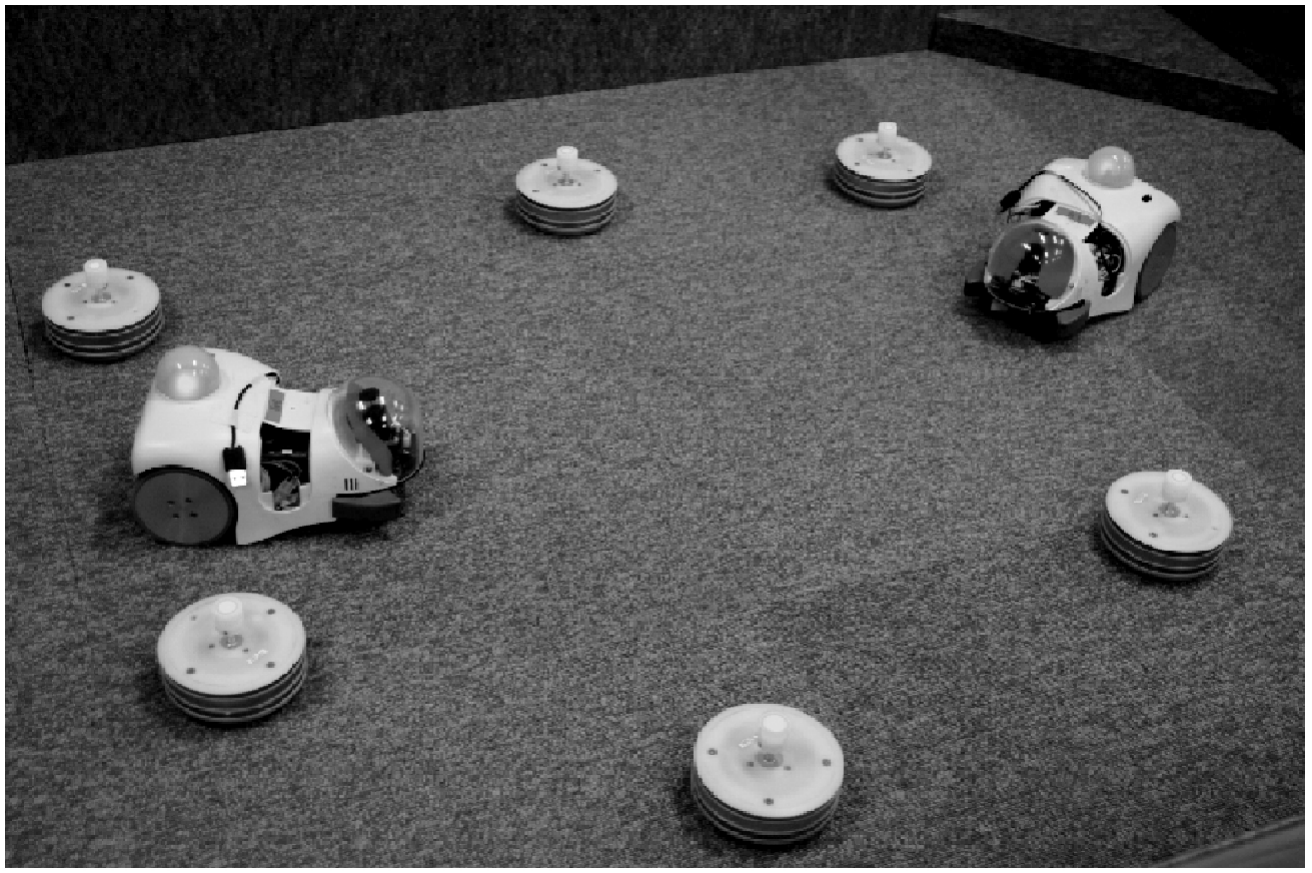
Two physical robots with six energy sources. The Cyber Rodent robots used in the experiments were equipped infrared communication for the exchange of genotypes and cameras for visual detection of energy sources (blue), tail-lamps of other robots (green), and faces of other robots (red).

**Figure 2:**
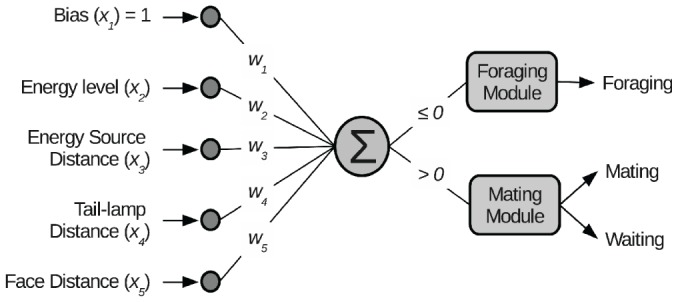
The neural network controller. The control architecture consisted of a linear artificial neural network. The output of the network was the weighted sum (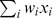) of the five network inputs (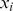) and the five evolutionarily tuned neural network weights (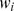). In each time step, if the output was less or equal to zero then the foraging module was selected, otherwise the mating module was selected. The basic behaviors were learned from by reinforcement learning with the aid of evolutionarily tuned additional reward signals and meta-parameters. The foraging module learned a foraging behavior for capturing energy sources. The mating module learned both a mating behavior for the exchange of genotypes, when a face of another robot was visible, and a waiting behavior, when no face was visible.

To investigate the ecological conditions for evolution of alternative mating strategies, we performed artificial evolution experiments, in simulation, with a small colony of Cyber Rodent robots [40] using our proposed embodied evolution framework [35]. We performed the experiments in simulation because of the infeasibility of running hundreds of generations of evolution in hardware. In previous work [35], we have shown that learned and evolved behaviors in simulation have similar performance and behavior when transferred to the hardware setting. Each individual interacted in small groups of four robots and during its lifetime of 288 seconds an individual experienced three periods of group interactions, where the participants in each group were randomly selected. We placed the four robots in an arena (2.5×2.5 m) with 4 to 16 energy sources. The robots were equipped with two wheels, an infrared port for the exchange of genotypes, and a camera that could detect energy sources, and tail-lamps and faces of other robots (Figure 1). The robots could execute three basic behaviors, foraging, waiting (for a potential mating partner), and mating, which were learned by reinforcement learning [41]. The mating strategy, i.e., the selection of basic behaviors, was controlled by a linear neural network and the (five) neural network weights were adapted by the evolutionary process (Figure 2). From a biological point of view, the population consisted of simultaneous hermaphrodites, who could reproduce offspring by mating (i.e., an exchange of genotypes with a mating partner). For each of the individuals involved in a mating event, the probability of reproducing offspring was linearly dependent on the individual's internal energy level (see Methods and [35]). This created a trade-off, where, in relative terms, an individual could maximize its fitness by maximizing either the frequency of mating events or the energy level at the mating events.

## Results

After the evolutionary process converged after 1,000 generations of experiments, we frequently observed a variety in the mating behavior when a robot could see both an energy source and the tail-lamp of a potential mating partner. We classified their mating strategies into two types: 1) *Forager strategy* in which an individual never waited for a potential mating partner and only tried to mate if it saw the face of another robot, and 2) *Tracker strategy* in which an individual waited for potential mating partner to turn around and where the threshold for waiting depended on its current energy level, the distance to closest energy source, and the distance to tail-lamp of the closest potential mating partner (in our preliminary report [42], we called them *roamer* and *stayer*, respectively, borrowing the terminology by Sandell and Liberg [43]). Among the high fitness populations (5 highest fitness populations in each of the 7 environments with different levels of energy source density), 31% (11/35) consisted predominantly of foragers and 49% (17/35) consisted predominantly of trackers. In 20% (7/35) of the populations, and remarkable four of the seven highest fitness populations, there emerged a polymorphic population of foragers and trackers with distinct differences in genotype, phenotype, and behavior. Our analyses show that the polymorphic population could constitute an ESS with an evolutionarily stable state of approximately 25% foragers in the population. In one instance, the trackers were split into two subpopulations: trackers who almost always waited for potential mating partners (strong trackers), and trackers who only waited if the energy level was high and an energy source was close (weak trackers). The analyses show that a population consisting of three phenotypes (foragers, strong trackers, and weak trackers) also could constitute a globally stable polymorphic ESS with several attractors.

**Figure 3:**
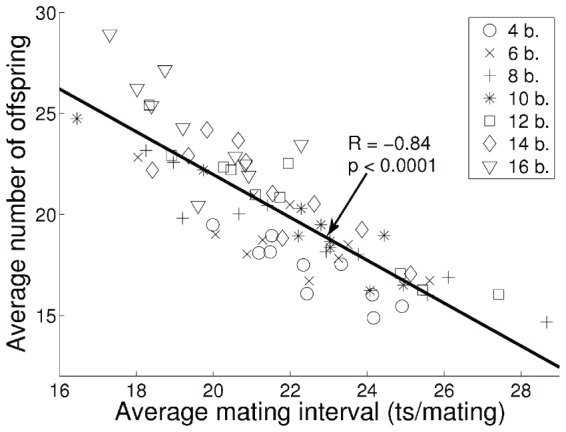
The correlation between the average estimated learning performance (i.e., the average mating interval) and the fitness (i.e., average number of offspring) in the final 20 generations in all experiments. The learning performance was estimated as the number of time steps the mating behavior was selected divided with number of mating events. The seven types of markers indicate the number of energy sources in the environment for each simulation.

The basic behaviors (foraging, waiting, and mating) of each individual were learned from scratch in each generation by reinforcement learning and accelerated by the evolution of the shaping rewards. For a detailed description and analysis of the evolution of shaping rewards and meta-parameters in our embodied evolution framework, see [35]. The fitness measured by the number of reproduced offspring correlated significantly with the learning performance of the mating behavior (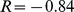 and 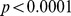; Figure 3). The efficiency of the mating behavior depended strongly on the evolved shaping reward functions. On average, one time step (240 ms) reduction in the time to execute a successful mating lead to an increase in fitness by approximately one offspring.

The purpose of performing experiments with different number of energy sources in the environment was to investigate the effect of energy source density on the emergence of different mating strategies. In general, higher energy source density resulted in higher energy levels at the mating events, which increased the probability of reproducing offspring and, therefore, the fitness (see Figure 3). However, we could not find an effect of energy source density on the evolution of different mating strategies.

### Analysis of Mating Strategies

**Figure 4:**
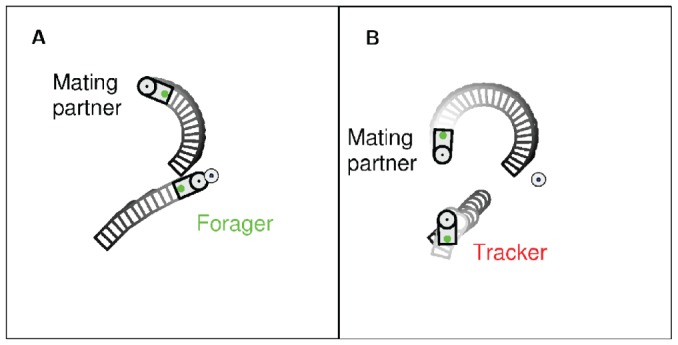
Example trajectories of the learned behaviors for the forager strategy and the tracker strategy. A) The forager ignores the tail-lamp of the mating partner and executes the learned foraging behavior to capture the energy source. B) The tracker executes the learned waiting behavior and adjusts its position according to the trajectory of the mating partner.

Analyses of the behaviors in the final 20 generations of the evolutionary process show that almost all individuals in all experiments executed an opportunistic behavior when either only an energy source was visible or only a face of another robot was visible. In the former situation all individuals always executed the foraging behavior and in the latter situation they always executed the mating behavior. An interesting case happened when a robot could see both an energy source and the tail-lamp of another robot from its behind. While some robots (i.e., foragers) chose to take the foraging behavior of approaching the energy source, others (i.e., trackers) took the mating behavior of keeping track of the tail-lamp and approaching when the face became visible, as illustrated in Figure 4. The choice in general depended on the robot's stored energy level and the distances to the energy source and the tail-lamp.

In order to characterize these behavioral phenotypes, we took the average energy level threshold 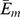 for the mating behavior, by computing the mean value over 676 equidistant visual inputs of the energy source and the tail-lamp (see Methods). With this criterion, the individual mating strategies emerged in the experiments could be classified: *Forager strategy*, in which an individual always took foraging behavior with 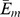 equal or very close to the maximum threshold (1), and 2) *Tracker strategy*, in which an individual waited for the potential mating partners to turn around when its energy level is above a certain threshold, with 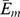 smaller than 1.

We identified the three different types of populations by computing the median and the standard deviation of the average threshold 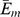 for all 1600 individuals in the last 20 generations in each of the 70 experiments. The populations with median values larger than 0.98 were considered forager populations. The remaining populations were identified by their standard deviations. Those with standard deviations smaller than 0.19 were considered tracker populations and those with standard deviations larger than 0.19 were considered polymorphic populations, all of which had multimodal 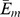 distributions.

We found 33, 28, and 9 populations of forager, tracker, and polymorphic strategies, respectively in the 70 populations. If we focus on the higher fitness populations (5 highest fitness populations in each of the 7 environments with different levels of energy source density), 31% (11/35) were foragers, 49% (17/35) were trackers, and 20% (7/35) were polymorphic. Remarkably, four of the seven highest fitness populations were polymorphic.

### Analysis of Genotypes, Phenotypes, and Behaviors

**Figure 5:**
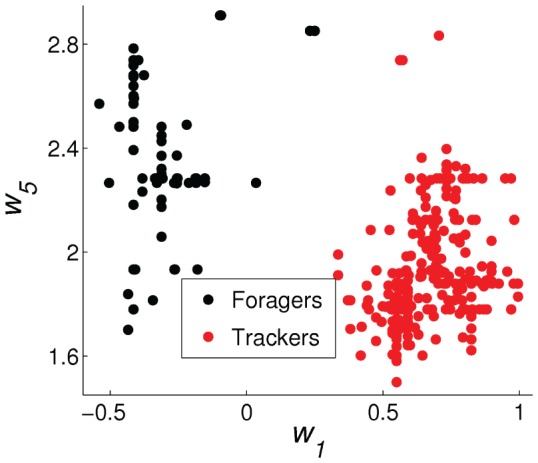
Difference in genotype between the forager and the tracker subpopulations. The distribution of values of the bias weights (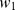) and the face distance weights (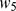) for all 1600 individuals in the final 20 generations.

**Figure 6:**
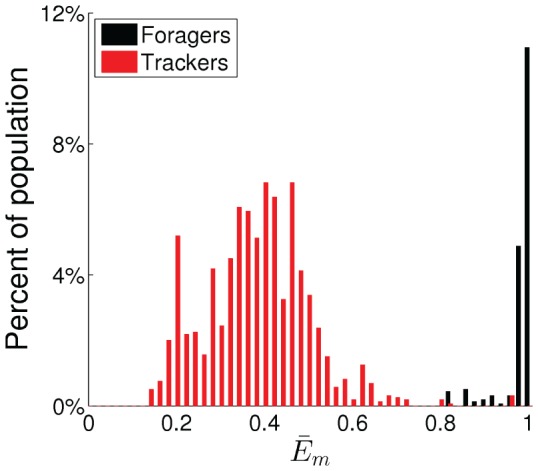
Difference in phenotype between the forager and the tracker subpopulations. The histograms of average waiting threshold values, 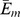, for all 1600 individuals in the final 20 generations.

**Figure 7:**
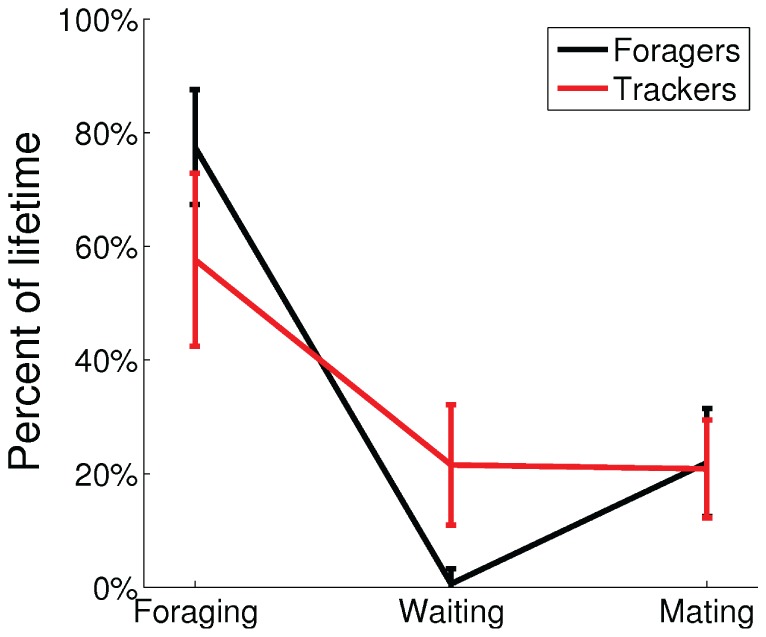
Difference in behavior between the forager and the tracker subpopulations. The mean percentages of the lifetimes, with standard deviation, the individuals spent executing the three basic behaviors for all 1600 individuals in the final 20 generations.

The best example of an emerged polymorphic mating strategy is shown in Figures 5–7, which emerged in the experiment with the highest average fitness in the environment with four energy sources. Figure 5 shows the distribution of the values of 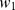, the bias for the mating behavior, and 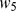, the weight for the distance to another robot's face, of all 1600 individuals in the final 20 generation. We classified the population into the two subpopulations by *k*-means clustering in the 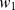 and 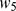 weight space and show the forager subpopulation in black and the tracker subpopulation in red in this and the following figures. Figure 6 shows the histogram of average energy thresholds 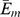 of selection of the mating behavior. The genetically classified foragers and trackers formed clearly distinct distributions. Figure 7 shows the average percentage of the lifetimes executing the three basic behaviors of foraging, waiting (choice of mating despite the face is not visible), and mating.

The result clearly shows a polymorphic population with distinct bimodal distributions in genotype, phenotype, and behavior for a forager subpopulation and a tracker subpopulation. For the foragers, the median of average energy threshold 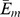 was equal the maximum threshold of 1. They spent on average 77.5% of their lifetimes executing the foraging behavior and almost no time (0.6%) executing the waiting behavior. In comparison, for the trackers the median of threshold 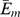 was 0.40. They spent on average 57.6% of their lifetimes executing the foraging behavior and 21.5% executing the waiting behavior.

### Evolutionary Stability of Polymorphism

**Figure 8:**
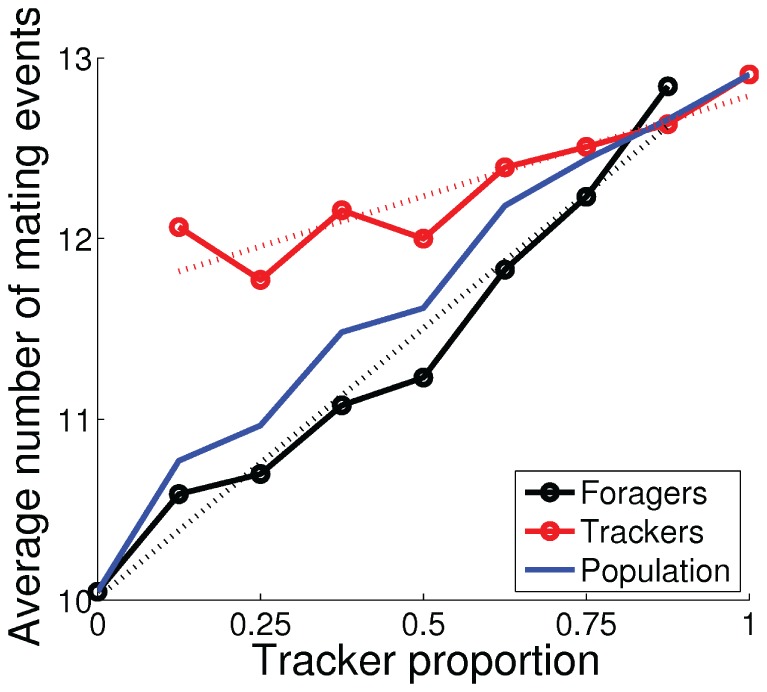
Average number of number of mating events as functions of the tracker proportion in the population. The dotted lines show the best linear fit for the two subpopulations and the blue line shows average values for the population as a whole.

**Figure 9:**
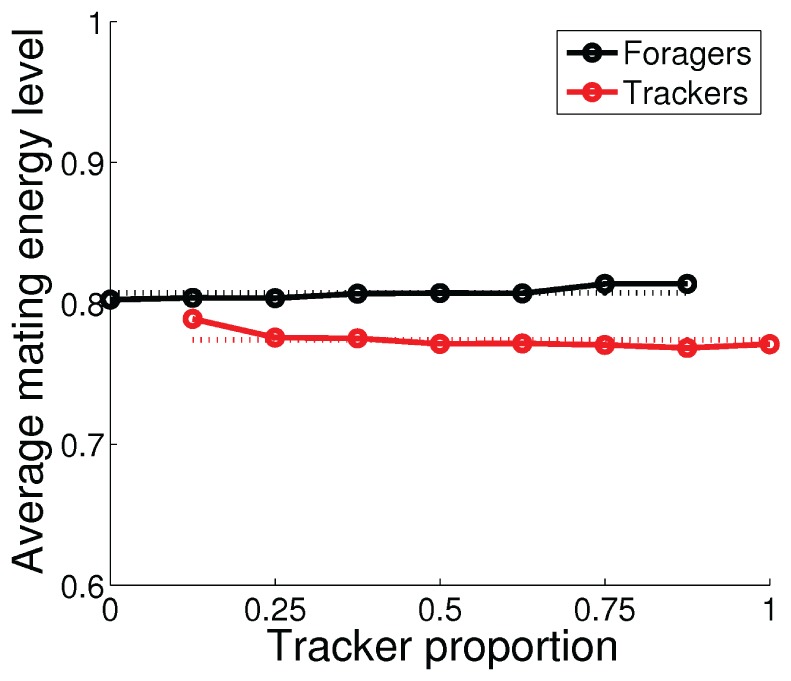
Average energy level at the mating events as functions of the tracker proportion in the population. The dotted lines show the constant approximations as the average values over all phenotype proportions.

**Figure 10:**
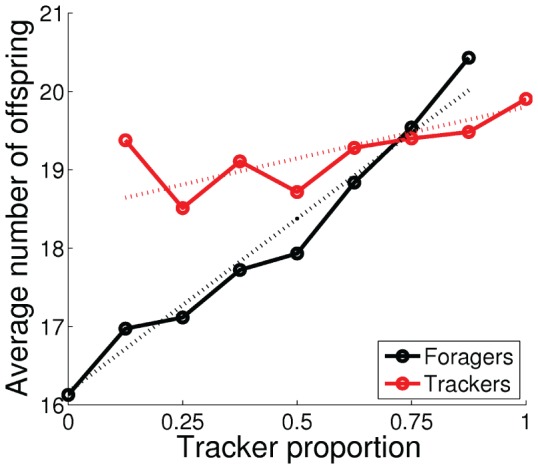
Average number of offspring (i.e., the fitness) as functions of the tracker proportion in the population. The dotted lines show the estimated fitness values using Equations 7 and 9.

To investigate the evolutionarily stability of the emerged polymorphic population of foragers and trackers, we performed additional experiments in which we fixed the proportion of the two phenotypes in the polymorphic population shown in Figures 5–7. Individuals from the two genotypes were selected randomly from the final (1000th) generation of the evolutionary experiment, which consisted of 10 foragers and 70 trackers. The experiment was repeated 100 times for each proportion of the two phenotypes. Figure 8 shows the average number of mating events, Figure 9 shows the average normalized energy level at the mating events, and Figure 10 shows the average number of offspring (i.e., the fitness) for the two phenotypes, as functions of the tracker proportion in the population. The number of mating events increased, both for the population as a whole and for the two phenotypes, as the number of trackers in the population increased. The increase in the number of mating events was much larger for the foragers (black line in Figure 8), from 10 to almost 13 mating events, as the tracker proportion increased from 0% to 87.5%. In comparison, the number of mating events for the trackers (red line in Figure 8) increased with only approximately 1 mating event, from 12 to 13, as the tracker proportion increased from 12.5% to 100%. The average energy levels were approximately constant over all phenotype proportions for both foragers and trackers (dotted lines in Figure 9) with a mean value of 0.81 for the foragers and 0.77 for the trackers. The experimental result clearly show (Figure 10) that the emerged population of foragers and trackers constitute a polymorphic ESS with an evolutionarily stable state of around 25% foragers in the population. The stable state corresponded to group-interactions with only one forager in the environment where the three trackers would all wait and adjust their trajectories according to the forager's behavior.

The foragers demonstrated negative frequency-dependent selection, i.e., the fitness increased as they became rarer. The foragers could be considered as a parasitic phenotype, because they relied on a high proportion of trackers to achieve a large number of mating events and, thereby, high fitness. In relative terms, the foragers tried to maximize the second term of the fitness function (Equation 1) by maximizing their own energy at the mating events, 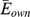. The trackers demonstrated positive frequency-dependent selection, i.e., the fitness increased as they became more common. However, the proportion of trackers in population had much less effect on the trackers' fitness. The average fitness increased only about 8% as the tracker proportion increased from 25% to 100%, compared to the foragers that increased their average fitness 21% as the tracker proportion increased from 0% to 75%. In relative terms, the trackers maximized the first term of the fitness function, the mating frequency 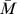, by using the waiting behavior to attract potential mating partners. The result also shows that the evolved mating strategy could have a significant impact on the average population fitness. There was an almost 4 offspring increase in fitness between a population with only foragers (fitness of about 16 offspring) and a population with only trackers or an evolutionarily stable polymorphic population (fitness of about 20).

### Mating Dynamics of Foragers and Trackers

Now let us investigate the dynamic interactions of the two subpopulations behind the observed fitness curves in Figure 10.

The average fitness 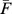, i.e., the number of reproduced offspring, is a function of the average number of mating events, 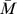, the average normalized energy level at the mating occasions (for offspring created by the individual), 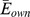, and the mating partners' average normalized energy level (for offspring created by the mating partners), 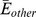:
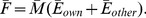
(1)


In order to model the average fitness, we take the following hypotheses: H1) The trackers achieved almost perfect mixing of mating partners. H2) The foragers mated more frequently with trackers than the proportion 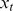 of trackers in the population. H3) The mating frequencies of the foragers, 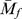, and the trackers, 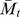, were functions of the tracker proportion 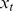. Under these conditions, we predict the fitness curves of the two subpopulations.

Let us denote the average numbers of mating events in a lifetime of a forager with foragers by 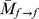, with trackers by 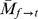, and with either by 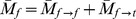. We also denote the average numbers of mating events of a tracker 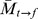, 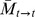, and 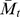 in the same convention.

**Figure 11:**
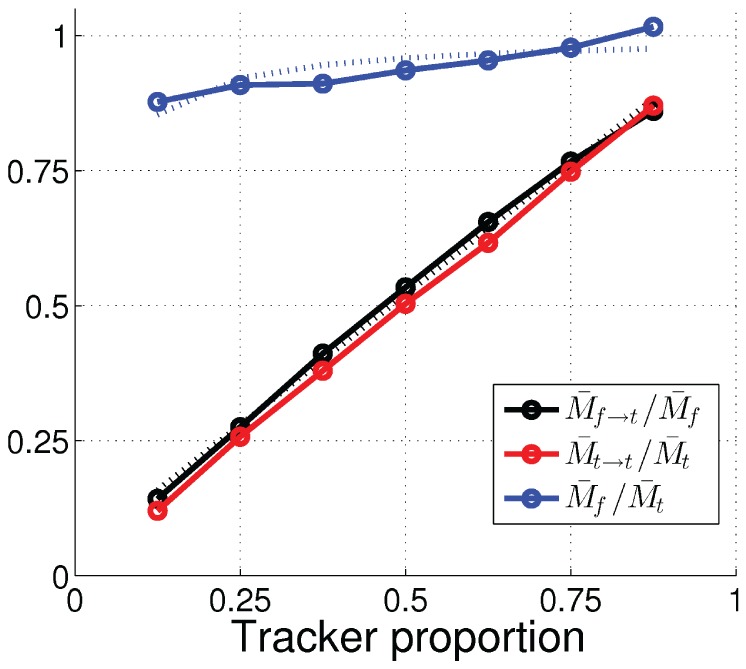
Average proportion of mating events with tracker mating partners as functions of the tracker proportion in the population, for the forager (black solid lines with circles) and the tracker (red solid lines with circles) subpopulations, and the ratio of average number of forager mating events to average number of tracker mating events (blue solid lines with circles). The black and red dotted lines show the best linear fit for the two subpopulations. The blue dotted line shows the predicted ratio of forager mating events to tracker mating events from Equation 5 with 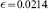.

H1 can be represented as
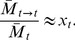
(2)This was true for all tested phenotype proportions, as shown by the red line in Figure 11.

H2 means that
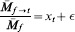
(3)with a positive 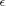. This was the case in our experiment with 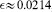, as shown by the black line in Figure 11.

From H1 (Equation 2) and the symmetry condition 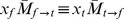, we have 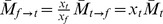. From this and H2 (Equation 3), the ratio of the average numbers of mating events by foragers and trackers (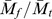) can then be derived as
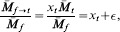
(4)

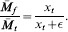
(5)In our experiment, the ratio 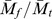 of forager mating events to tracker mating events increased from 0.88 to 1.02 when the tracker proportion 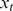 increased from 0.125 to 0.875, as shown by the blue line in Figure 11. This fits well with the model prediction in Equation 5 with 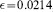, shown by the dotted blue line in Figure 11.

From H3, the average fitness (Equation 1) of the foragers, 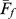 and the trackers, 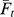, can be rewritten as
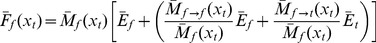
(6)

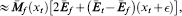
(7)

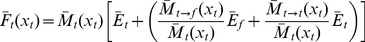
(8)

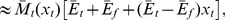
(9)The dotted lines in Figure 10 shows the estimation of the average fitness of the foragers and the trackers, using Equations 7 and 9, respectively, with constant 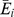-values, and with the best linear fit of 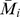 from the data (see dotted lines in Figures 9 and 8, respectively).

### Population with Three Mating Strategies

**Figure 12:**
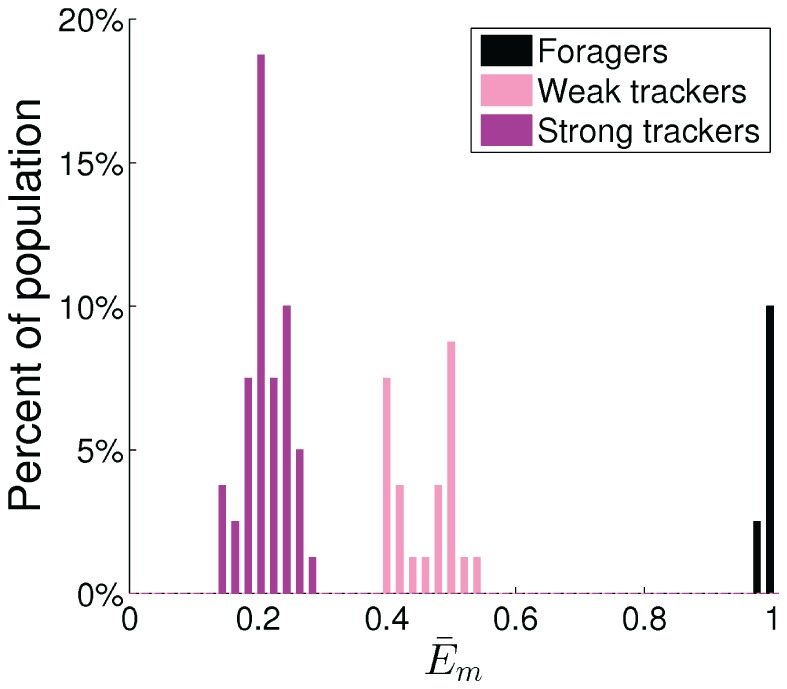
Difference in phenotype between the forager (black), the weak tracker (pink), and the strong tracker (purple) subpopulations. The histograms of average waiting threshold values, 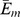, in the final generation.

**Figure 13:**
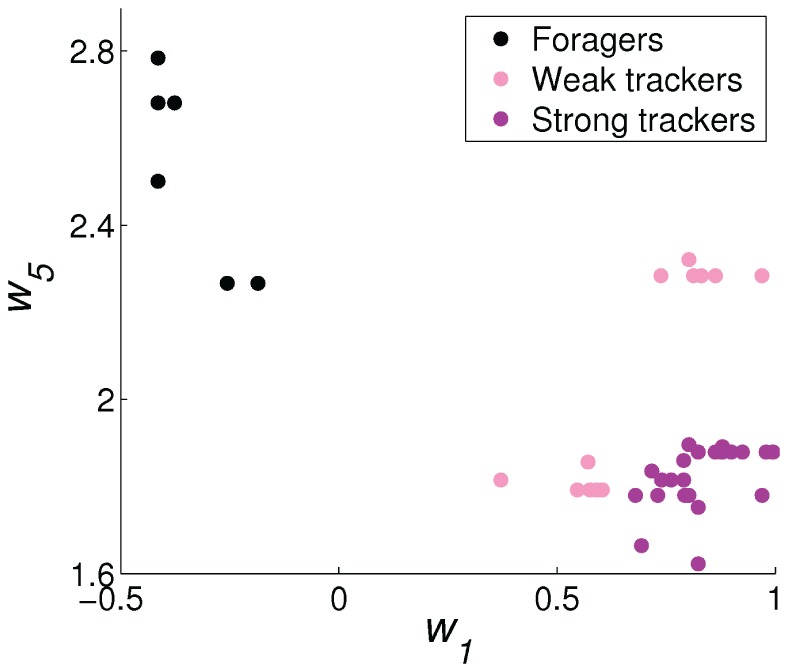
Difference in genotype between the forager (black), the weak tracker (pink), and the strong tracker (purple) subpopulations. The distribution of values of the bias weights (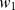) and the face distance weights (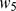) in the final generation.

As shown in Figure 6, the distribution of the average energy threshold for waiting, 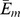, of all trackers in the final 20 generations was very broad. Interestingly, in the final generation, the trackers' phenotype distribution was bimodal with two distinct peaks (Figure 12): a larger subpopulation (46 individuals) of *strong trackers* with a smaller median 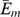-value of approximately 0.22 and a smaller subpopulation (24 individuals) of *weak trackers* with a larger median 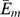-value of approximately 0.48. The separation in genotype between the strong and weak trackers was not as distinct, but it is clearly visible in 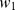 and 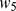 weight space (Figure 13).

**Figure 14:**
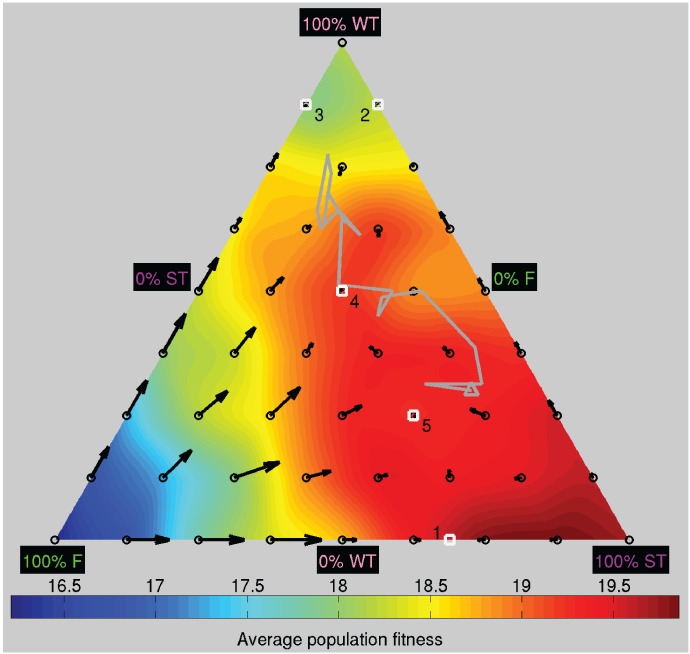
DiFinetti diagram of the directions and magnitudes of the changes in subpopulation proportions for the three phenotypes: foragers (F), weak trackers (WT), strong trackers (ST). The small black circles indicate the tested phenotype proportions. The black arrows show the average direction and magnitude of the change in phenotype proportions (magnified by a factor of five for visualization purposes). The white numbered squares indicate the approximate population ratios of the five evolutionary stable states, where the ratios of the phenotypes (F, WT, ST) were 1: (31.25%, 0%, 68.75%), 2: (0%, 87.5%, 12.5%), 3: (12.5%, 87.5%, 0%), 4: (25%, 50%, 25%), and 5: (25%, 25%, 50%). The gray line, ending in the gray triangle, shows the phenotype proportions in final 20 generations of the evolutionary experiments. The background coloring visualizes the average population fitness.

To investigate the evolutionarily stability of a population consisting of foragers, weak trackers, and strong trackers, we conducted additional experiments in which we fixed the proportions of the three phenotypes and measured their fitnesses. As in the earlier experiments, the genotypes of the three subpopulations were randomly selected from the genotypes in the final generation of the evolutionary experiments and repeated 100 times for each proportion of the three phenotypes. The result of the experiments is summarized in the DiFinetti diagram in Figure 14. The proportion of each phenotype increases from the side of the triangle to the opposite vertex. The black circles represent the tested phenotype proportions. In general, the average population fitness (shown by the background coloring of the diagram) increased with the number of strong trackers in the population. The average population fitness was 16.2 offspring in population with only foragers, 18.2 offspring in population with only weak trackers, and 19.7 offspring in a population with only strong trackers, which was close to the maximum average fitness of 19.9 for a population consisting of 87.5% strong trackers and 12.5% foragers.

Assuming a fixed population size, the proportion of phenotype *i* in the next generation, 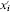, can be calculated by the discrete replicator dynamic equation:
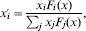
(10)where, 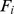 is the average fitness of phenotype *i* with proportion 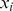 of the population in the current generation. The black arrows in Figure 14 show the average directions and magnitudes of the changes in phenotype proportions. Three populations consisting of only two of the phenotypes (as seen along the three sides of the triangle) were evolutionarily stable with populations proportions close to: 1) 31.25% foragers and 68.75% strong trackers (bottom side); 2) 87.5% weak trackers and 12.5% strong trackers (right side); and 3) 12.5% foragers and 87.5% weak trackers (left side). Populations consisting of all three phenotypes were globally stable, because all arrows in the inner triangle, representing populations consisting of at least 12.5% of each of the three phenotypes, point inside the triangle. The diagram suggests that there were two evolutionarily stable attractors where the magnitude of the change in the proportion of the phenotypes was zero, 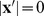, with centers close to populations consisting of: 4) 25% foragers, 50% weak trackers, and 25% strong trackers (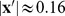) and 5) 25% foragers, 25% weak trackers, and 50% strong trackers (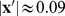). For populations with 12.5% to 25% foragers, the average magnitudes of the change in phenotype proportions were small, i.e., low selection pressure, which meant that phenotype ratios could move relatively easily between the attractors by random genetic drift. For example, in the final 20 generations of the evolutionary experiment (illustrated by the gray line ending in the gray triangle), the phenotype ratios were first close to the fourth attractor (fitness of 19.1), and ended up close to the fifth attractor (fitness of 19.3).

## Discussion

In this study, we demonstrated that polymorphic mating strategies can emerge in a small robot colony under homogeneous evolutionary conditions, without a selection scheme or an explicit fitness function that promoted a certain outcome. Our study is, to our knowledge, the first to demonstrate the emergence of polymorphic ESSs within a robot evolution framework. This gives further evidence that artificial robot evolution (for an overview see [28]) can be a feasible and a valuable approach for investigating hypotheses of biological phenomena.

The importance of specific details of the genetic algorithm and the structure of the genotype were illustrated in this study. A condition for the evolution of the polymorphic ESSs consisting of foragers and trackers was the small proportion of genotype controlling the mating strategy, in combination with the relatively low crossover rate. The mating strategy was controlled by only 5 out of the 51 genes, located at the beginning of the genotype, and with a crossover rate of 0.1 there was only a 0.8% probability that an offspring would have a mating strategy controlled be a mixture of genes from both parents. A much more frequent mixing of the mating strategy genes would have made it more difficult or even impossible to evolve and maintain separate genetic traits in the same population. An assumption underlying evolutionary game theory [7], is that the payoffs that agents are assumed to be without noise. It is therefore very encouraging that evolutionarily stable polymorphic ESSs could emerge in our experiment with a small population size and with large variances in the performance of similar, and even identical, individuals. The lifetime learning of the basic behaviors by reinforcement learning introduced additional stochasticity. Even in the last part of the evolutionary process, a few individuals failed to capture any batteries or engage in any mating activity. This was usually caused by that the individual got trapped in a corner of the environment and failed to learn how to navigate out of it.

The forager strategy in the evolved polymorphic populations can be seen as a cheater strategy. To achieve high fitness, a forager relies on that all the other individuals in the environment (i.e., trackers) will adjust their behaviors according to the trajectory of the forager. The forager, therefore, avoids the cost of searching for mating opportunities. There exists a rich literature on the potential of cheating in hermaphrodite mating systems (for an overview see [44]). Usually, cheating refers to the attempt of individuals to take on the male role over the female role in mating encounters to avoid the cost offspring reproduction.

The most similar study to ours was conducted by Rold *et al.* [45]. They co-evolved a population of predefined male and female robots. The robots, as in our experiments, remained alive by capturing energy sources and reproduced by physical mating, consisting of touching a robot of the opposite sex. The only difference between males and females was that the males remained reproductive throughout their lives, while the females became non-reproductive for a fixed period of time after an reproductive mating event. In their experiment, the reproduced offspring were not the result of a genetic exchange between mating robots. Instead, the males and females were evolved separately with the number of reproductive mating events used as fitness objective. The evolved behaviors of the males and females had distinct differences and their behaviors corresponded to observed behaviors of males and females in biological studies. Interestingly, the evolved behaviors of the males and females also matched the behavior of the foragers and trackers, respectively, in our study. The males opportunistically ate all the food they could find while looking for reproductive females. The reproductive females were less active and adopted a mating strategy of waiting for males to mate with them. This give some support to a hypothesis that polymorphic mating strategies, emerged due to basic trade-off between the resources spent on energy conservation and the resources spent on courtship of mating partners, is a precursor of sexual dimorphism. In our experiments, polymorphism could arise because the foragers and trackers optimized, in relative terms, different parts of the fitness function (Equation 1). The foragers maximized their own energy level, 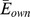, by spending all their lives foraging for energy sources except for when a a potential mating partner was directly visible, while the trackers maximized the mating frequency, 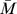, by spending considerable amount of their lives waiting for potential mating partners. The evolution of “proto-sexes” is a research venue we plan to explore in future work. In the current experimental setup, both the sender and receiver can reproduce offspring at the mating events and the cost of mating is equal, whether offspring are reproduced or not (see *Methods*). A more biological plausible setup would be that only one of the agents took on the female role, e.g., the receiver, and also bore the main cost of reproducing offspring. The goal would then be to investigate if, and in such case under which conditions, a breeding system with distinct male and female roles could evolve from an initial population of hermaphrodites without any predefined mating preferences, and maybe even more exotic breeding systems such as *androdioecy* (males and hermaphrodites) and *gynodioecy* (females and hermaphrodites).

## Methods

Four Cyber Rodent mobile robots [40] were placed in a 2.5×2.5 m arena, together with 4, 6, 8, 10, 12, 14, or 16 energy sources (Figure 1). The task of the robots were to survive by maintaining their internal energy level through foraging of energy sources and by reproduction of offspring through physical exchange of genotypes by infrared communication. We performed the experiments in a simulation environment, developed to mimic the features of the real Cyber Rodent hardware platform. The robots were equipped with a camera system with color blob detection, used to extract the distances and relative angles to nearest energy source (blue), the nearest tail-lamp of another robot (green), and the nearest face of another robot (red). Mimicking the real robotic hardware, the field of view of the simulated vision system set to 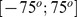. Within an angle range of 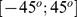, the robots could detect energy sources up to 2 m, tail-lamps up to 1.5 m, and faces up to 1 m. Outside this range, the detection capability decreased linearly down to 0.2 m for the maximum angles.

We performed 1000 generations of evolution and for each energy source density, we ran 10 evolution experiments. To be able to conduct robot evolution experiments with only only four robots, we utilized time-sharing in subpopulations of 20 individuals within each robot. Each individual in a subpopulation took control, in random order, of the robot for three time-sharings of 400 time steps, i.e., the total lifetime was 1200 time steps. An individual had a maximum internal energy level (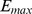) of 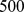 energy units. Each time step, the energy level decreased by 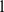 unit and a capture of an energy source increased the energy level by 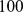 units. At birth, an individual had an internal energy level of 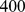 units. If an individual's energy was depleted, then the individual died and was removed from its subpopulation. When a robot captured an energy source it disappeared from its current position and reappeared in new, randomly selected, position.

We did not apply an explicit fitness function or a centralized selection process, instead offspring were created by a mating. The individuals controlling the robots could create offspring by a physical exchange of genotype through infrared communication. The infrared communication ports were located slightly to the right of center in the front of the Cyber Rodent robots, directed straight forward. In the simulation environment, the maximum range of the communication was set to 1 m and the angle range was set to 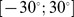. An individual could initiate the infrared communication by executing a predefined action selected by the mating behavior. For a mating event to be successful, both robots had to be within each others mating range before and after the individuals controlling the robots executed the actions of their currently selected reinforcement learning modules. The probability, for each of the two individuals involved in a mating event, of reproducing offspring was linearly depended on the individual's energy level (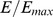). A reproductive mating event created two offspring in the individual's subpopulation by applying one-point crossover with a probability of 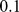. The genes of the two newly created genotypes were then mutated with probability of 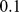, by adding value from a Gaussian distribution with zero mean and a standard deviation of 0.1. After all individuals in a subpopulation had survived for a full lifetime or died a premature death, a new subpopulation was created by randomly selecting a fixed number, i.e., 20 in our experiments, of the offspring reproduced during the last generation.

The genotype consisted of 51 real-valued genes: 1) 5 genes controlling the mating strategy by encoding the weights of the top-level neural network that selected basic behaviors (Figure 2); 2) 42 genes determining the parameters of the additional reward signals for the basic behaviors in the form of potential-based shaping rewards [46]; and 3) 4 genes determining the meta-parameters of the reinforcement learning algorithm. The five-dimensional input to the neural network consisted of a constant bias of 1 (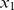), the individual's internal energy (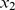), and the inverse distances to the nearest energy source (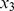), tail-lamp (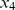), and face (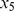). The sensory inputs were linearly scaled to a range of 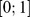. If a visual target was not visible, the corresponding input value was set to −1. In each sensory-motor cycle (time step), the output of the neural network (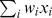) determined which of two reinforcement learning modules that was selected. If the output was greater than zero the mating module was selected, otherwise the foraging module was selected. After a successful mating event, whether it reproduced offspring or not, an individual could not select the mating module again until it had captured an energy source or until 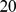 time steps had passed. During this time, the tail-lamp was turned off.

In the case when only an energy source and a tail-lamp were visible, the energy thresholds for the selection of the mating module, 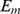, was given by
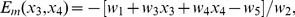
(11)which depended on the distance to the closest energy source (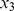) and the distance to the closest tail-lamp (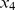). In order to derive the average energy threshold 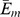, we computed the mean of 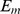 over 676 values of 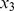 and 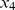 (26 equidistant values between 0 and 1 for each of the two sensory inputs).

The reinforcement learning modules learned their behaviors from scratch in each generation with the aid of evolutionarily tuned potential-based shaping rewards and meta-parameters. The foraging module executed a foraging behavior using the relative angle and the distance to the nearest energy source as state variables. The mating module executed either a mating behavior or a waiting behavior, depending on the current sensory inputs. If a face of another robot was visible, the mating behavior was executed using the relative angle and the distance to the nearest face as state variables. Otherwise, the waiting behavior was executed using the relative angle and the distance to the nearest tail-lamp as state variables. The behaviors were learned by the Sarsa reinforcement learning algorithm [47], [48] with tile coding [48] and potential-based shaping rewards [46]. The global reward for the reinforcement learning modules was set to +1 for a successful mating event and +1 for a capture of an energy source, otherwise the reward was set to 0.

The additional experiments, conducted to investigate the evolutionarily stability of the emerged polymorphic ESSs, were performed in a similar manner as the evolution experiments. The only difference was that, in each generation, the subpopulations were created by randomly selecting the genotypes of the different phenotypes from the final generation of the evolution experiment according to the predefined phenotypes ratios.

For a detailed description of our embodied evolution framework and algorithm specifics, see [35].
